# Factors predicting hospital length-of-stay and readmission after colorectal resection: a population-based study of elective and emergency admissions

**DOI:** 10.1186/1472-6963-12-77

**Published:** 2012-03-26

**Authors:** Maria Kelly, Linda Sharp, Fiona Dwane, Tracy Kelleher, Harry Comber

**Affiliations:** 1 National Cancer Registry, Building 6800, Cork Airport Business Park, Cork, Ireland

**Keywords:** Colorectal, Cancer, Length-of-stay, Surgery, Elective, Emergency, Readmission rate

## Abstract

**Background:**

The impact of developments in colorectal cancer surgery on length-of-stay (LOS) and re-admission have not been well described. In a population-based analysis, we investigated predictors of LOS and emergency readmission after the initial surgery episode.

**Methods:**

Incident colorectal cancers (ICD-O2: C18-C20), diagnosed 2002-2008, were identified from the National Cancer Registry Ireland, and linked to hospital in-patient episodes. For those who underwent colorectal resection, the associated hospital episode was identified. Factors predicting longer LOS (upper-quartile, > 24 days) for elective and emergency admissions separately, and whether LOS predicted emergency readmission within 28 days of discharge, were investigated using logistic regression.

**Results:**

8197 patients underwent resection, 63% (n = 5133) elective and 37% (n = 3063) emergency admissions. Median LOS was 14 days (inter-quartile range (IQR) = 11-20) for elective and 21 (15-33) for emergency admissions. For both emergency and elective admissions, likelihood of longer LOS was significantly higher in patients who were older, had co-morbidities and were unmarried; it was reduced for private patients. For emergency patients only the likelihood of longer LOS was lower for patients admitted to higher-volume hospitals. Longer LOS was associated with increased risk of emergency readmission.

**Conclusions:**

One quarter of patients stay in hospital for at least 25 days following colorectal resection. Over one third of resected patients are emergency admissions and these have a significantly longer median LOS. Patient- and health service-related factors were associated with prolonged LOS. Longer LOS was associated with increased risk of emergency readmission. The cost implications of these findings are significant.

## Background

There were over one million cases of colorectal cancer diagnosed worldwide in 2008. It is the third most common cancer in European populations and with population ageing, the number of new cases is expected to rise [1]. In Ireland just over 2000 cases were diagnosed in 2008 and the numbers are projected to increase by 45% in men and 34% in women between 2010 and 2020 [2].

Diagnosis and treatment of each case of colorectal cancer is estimated to cost around €40,000 with hospital care accounting for much of this [3,4]. Surgery remains the cornerstone of treatment, and hospital stay is likely to be an important contributor to costs.

Length-of-stay (LOS) is an indicator of health service efficiency. Various initiatives aimed directly at reducing LOS, such as enhanced recovery programmes, have been instigated in the USA and Europe [5-8]. In colorectal cancer specifically, there have been a range of other developments in surgery, including greater surgical specialization and wider use of laparoscopic procedures [9,10]. As well as potentially reducing LOS, these are intended to confer advantages for patients, including faster recovery and fewer complications. One of the key concerns of attempts to reduce LOS, however, is that it may compromise patient safety and lead to increased readmissions [11,12].

It was against this background that we conducted a population-based analysis of time trends in LOS and predictors of longer LOS following colorectal resection. Unlike previous studies (Faiz et al., 2010), we considered elective and emergency admissions in individuals with confirmed colorectal cancer, since the latter account for a significant proportion of patients. We further investigated factors predicting emergency readmission rates within 28 days of first admission and, specifically, whether LOS affected this.

## Methods

The study setting was Ireland, which has a mixed public-private health care system. All residents are entitled to use the public health system; this provides primary care services, hospital out-patient treatment, and in-patient treatment in public hospitals. Public hospitals also offer private health care, and patients can opt to transfer from public to private care. Finally patients can be treated in entirely private hospitals. Thus there are three categories of patients: (1) public patients treated within public hospitals (2) private patients who pay for treatment within public hospitals and (3) private patients treated in private hospitals.

The primary data sources for this study were the National Cancer Registry (NCR) and the Hospital In-Patient Enquiry Scheme (HIPE). The NCR records demographic, clinical and treatment information for all cancers diagnosed in the population usually resident in Ireland, according to internationally accepted registration and coding conventions http://www.ncri.ie. The majority (97.5%) of registrations are made actively by tumour registration officers (TROs) who collate and abstract data from various sources including pathology laboratories, radiotherapy clinics and medical records departments. Remaining registrations are from death certificates (2%) and general practitioners (< 0.5%). Death certificates are provided nationally by the Central Statistics Office http://www.cso.ie; dates of death are ascertained by linkage to death certificates using probabilistic matching methods. For all cancers (excluding non-melanoma skin cancer) diagnosed in 2003, the completeness of case ascertainment after five years of follow up is estimated to be 98% [13].

HIPE is a computer-based information system that collects data on discharges from all acute public hospitals in Ireland http://www.esri.ie/health_information/hipe. Demographic, clinical and administrative data are collected [14]. Data are subject to numerous computer-based edits/checks at data entry and later validation checks [15]. Private hospitals can volunteer to contribute data to HIPE, however coverage is incomplete and we limited our analysis to patients treated in public hospitals (as either public or private patients). The NCR is provided with all HIPE records which mention cancer in one of the diagnosis fields.

The NCR has permission under the Health (Provision of Information) Act 1997 to collect and hold data on all persons diagnosed with cancer in Ireland. The use of that data for research is covered by the Statutory Instrument which established the Registry Board in 1991. All datasets were anonymised prior to analysis.

**Figure 1:**
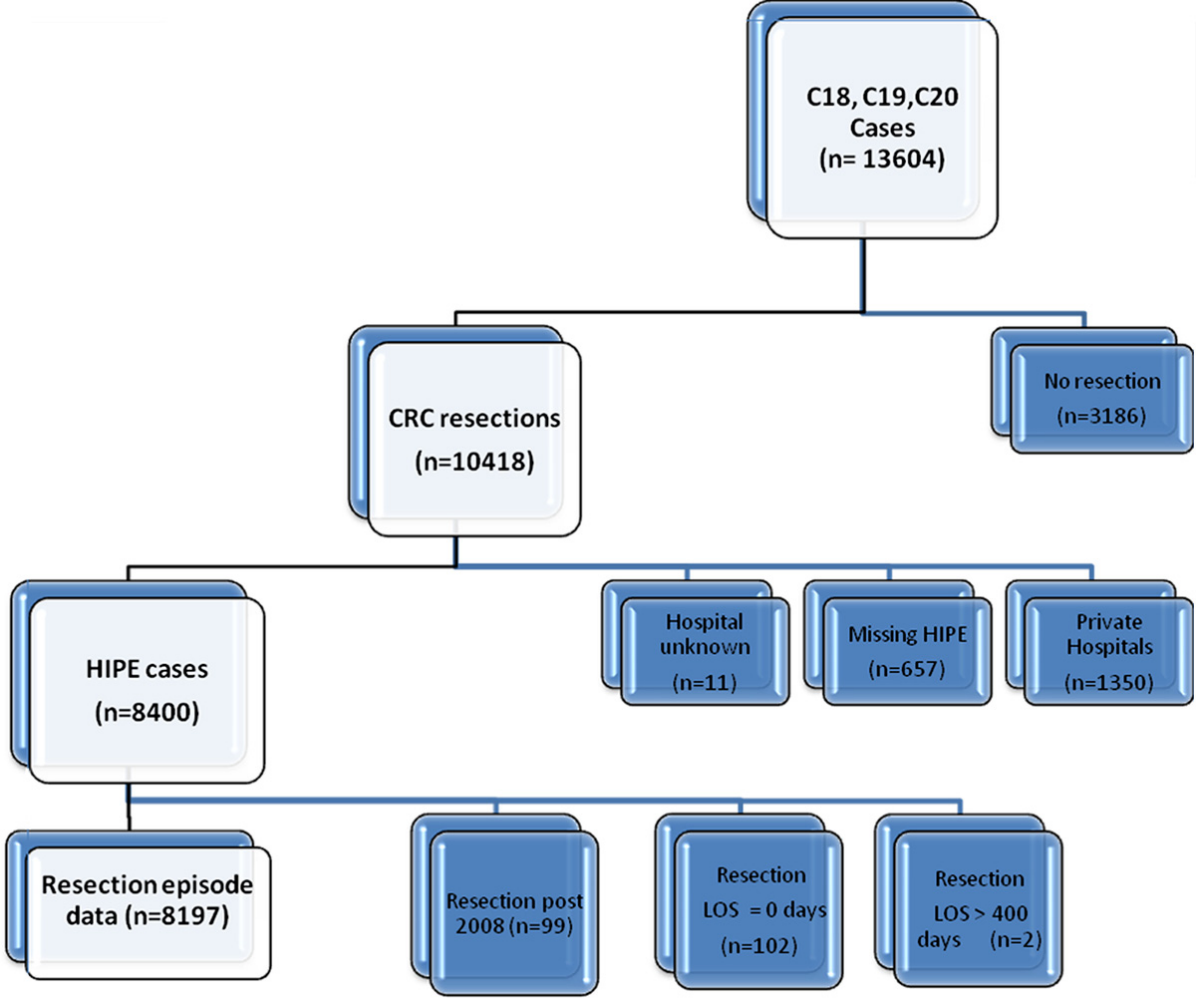
**Project overview**.

Colorectal cancer patients (ICD-O2: C18-C20) newly diagnosed between 2002 and 2008 were identified from the NCR. Individuals who had another primary cancer prior to the colorectal cancer (other than non-melanoma skin) were excluded. The dataset was then limited to those who had a colorectal resection according to NCR records, (ICD-9-CM codes 45.4x, 45.7x, 45.8, 48.3, 48.35, 48.36, 48.4, 48.49, 48.5, 48.6x, 48.82) [16]. Using probabilistic matching techniques, these patients were linked to HIPE episodes (Figure 1).

HIPE episodes were ordered by date of admission and overlapping episodes were combined. Each admission was classified as emergency or elective according to HIPE codes. Admissions are recorded as elective when the patient's condition permits adequate time to schedule accommodation and delays do not cause a substantial risk to health. Admissions are recorded as emergency when the patient requires immediate care and treatment as a result of a severe, life threatening or potentially disabling condition http://www.esri.ie/health_information/hipe.

The date of first colorectal surgery recorded by the NCR was matched to the corresponding HIPE episode. LOS was calculated as the number of days between admission and death or discharge. Duration of discharge was calculated as the time from the discharge following the index colorectal resection to the next admission (if any). Patients whose length of discharge was less than 29 days were considered readmissions. In the UK 28-day emergency re-admission rate is a key hospital performance indicator http://www.nchod.nhs.uk and has been used elsewhere [12].

The level of deprivation of each patient's area of residence (at diagnosis) was measured using an index created from 2002 census variables [17]. A co-morbidity score for each patient, based on the Charlson index, was derived from all diagnoses recorded in HIPE for the index surgery episode; the colorectal cancer diagnosis was disregarded in this calculation [18,19]. Cases were categorised according to whether they underwent surgery at a hospital located within or outside the health region in which they resided at diagnosis. The volume of colorectal cancers treated at each hospital and by each consultant was derived from NCR data and calculated as the median number of colorectal resections performed per year. Discharge status was obtained from HIPE; patients are classified as public or private to the consultant at the time of discharge. Stage at diagnosis was defined according to American Joint Committee on Cancer (AJCC) summary staging from NCR data [20]; cases where information on distant metastasis (MX) was not recorded were considered 'unknown' stage. Patients destination at discharge was classified as home, care (i.e. nursing home, convalescent home or long stay accommodation), death or other (i.e. transfer to hospital, psychiatric unit, prison, temporary residence, hospice, self-discharge or absconded).

### Statistical analysis

Analyses were conducted using Stata 11 [21]. Median LOS was computed overall and separately (a) by year, (b) for colon and rectal tumours, and (c) for emergency and elective admissions. Differences in LOS by year of surgery were examined using the Kruskal-Wallis equality-of-populations rank test [22] and Cuzick's non-parametric test for trend [23].

LOS was categorized into approximate quartiles based on all cases. Since we are not aware of any national, or internationally agreed, definition of prolonged LOS following colorectal resection, in the primary analysis we defined prolonged hospital stay as duration greater than the upper-quartile for all cases (> 24 days). Multivariable logistic regression was used to identify factors which predicted a prolonged hospital stay. Separate models were built for emergency and elective admissions. Three types of variables were considered for inclusion in the model: socio-demographic (age, gender, marital status, deprivation index, discharges status); clinical (tumour site, stage, co-morbidity) and care (admission type, hospital volume, consultant volume, health board of hospital, year of diagnosis). Subjects with missing data for any of these covariates were excluded from relevant analyses. Variables were included in the multivariable model if they were significant (p < 0.1) on likelihood ratio tests. Model goodness-of-fit was checked using the Hosmer and Lemeshow test [24]. We did a sensitivity analysis using the median as cut-off point for prolonged LOS (i.e. LOS > 16 days in our study) as has been used elsewhere [25,26].

The same approach was used to identify factors predicting emergency readmissions. For this analysis, patients were classified as (a) died during the index surgery episode, (b) died within 28 days of discharge, (c) emergency readmission within 28 days of discharge, or (d) not readmitted or elective readmission within 28 days. In the analysis, the cases included group (b) and (c) and controls were group (d). Once a "core" model had been built, LOS (in quartiles) was fitted to examine its association with readmission. We looked for a trend in readmission rates over time by fitting 'year of surgery' as a continuous variable.

## Results

10418 incident colorectal cancer patients who had undergone resection were identified from the NCR (Figure 1). 80.6% (n = 8400) had a corresponding HIPE episode; Those who had surgery in a private hospital (12.9%), or in a public hospital but who had no HIPE record (6.3%), or the hospital of treatment was unknown (0.11%) were excluded. Patients resected in 2009 (n = 99), day cases (n = 102) and patients with implausibly long LOS (> 400 days; n = 2) were also excluded, leaving 8197 cases available for analysis. Of these 37% (n = 3063) presented as emergency admissions.

**Figure 2:**
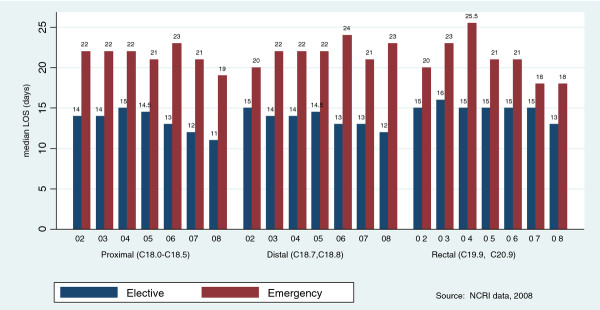
**Median LOS for all admissions by site, admission type and year of surgery**.

Nearly 95% of colon cancer patients having resection spent more than 7 days in hospital while over 80% of rectal cancers patients having a resection stayed more than 10 days. Table 1 describes median LOS for admissions by demographic, clinical and care variables. Median LOS was 16 days (inter-quartile range = 11-25 days) for all patients; 14 (IQR = 11-20) for elective admissions and 21(IQR = 15-33) for emergency admissions. Median pre-surgery LOS was 2 days (IQR = 1-4) for elective admissions and 6 days (IQR = 2-13) for emergency admissions. Median post-surgery LOS was 11 days (IQR = 8-16) for elective and 14 days (IQR = 10-21) for emergency admissions. Over the study period median LOS decreased significantly for proximal (C18.0-C18.5), distal (C18.7, C18.8) and rectal cancers (C19.9, C20.9) for elective admissions, (p < 0.001 for all three). For emergency admissions median LOS decreased significantly (p < 0.032) for proximal cancers but not for distal (p < 0.278) or rectal cancers (p < 0.086) (see Figure 2).

**Table 1:** Median (M) and inter-quartile (IQR) length-of-stay for colorectal cancer patients having resection 2002-2008

	Elective admissions (n = 5133)			Emergency admissions(n = 3063)		
	**n (%)**	**M**	**IQR**	**n (%)**	**M**	**IQR**

**Age at diagnosis**						

< 60	1311 (25.5)	12	10-16	608 (19.6)	16	12-23

60-69	1512 (29.5)	14	10-18	712 (23.3)	20	14-30

70-79	1637 (31.9)	15	11-21	1009 (32.9)	24	16-36

80+	673 (13.1)	18	13-28	734 (24.0)	27	18-39

Gender						

Male	3059 (59.6)	14	11-21	1644 (53.7)	21	15-32.5

Female	2074 (40.4)	14	11-19	1419 (46.3)	22	15-34

**Marital status**						

Married	3188 (62.4)	13	10-18	1582 (51.8)	20	14-29

Other	1925 (37.6)	15	11-22	1474 (48.2)	24	16-37

**Deprivation index**^**1**^						

1 (least deprived)	903 (17.6)	14	11-20	602 (19.7)	20	14-30

2	655 (12.8)	14	10-20	395 (12.9)	20	14-31

3	685 (13.3)	14	11-20	329 (10.7)	22	15-34

4	877 (17.1)	14	11-20	521 (17.0)	22	15-34

5 (most deprived)	1574 (30.7)	14	11-20	987 (32.2)	22	15-34

missing	439 (8.6)	14	11-20	229 (7.5)	21	15-32

**Smoking status**						

Ever	1909 (37.7)	14	11-21	1017 (33.8)	22	15-34

Never	2316 (45.7)	14	10-19	1273 (42.3)	21	15-33

Unknown	836 (16.5)	15	11-22	719 (23.9)	21	14-32

**Comorbidity**^**2**^						

0	4137 (80.6)	14	10-19	2267 (74.1)	20	14-30

1	700 (13.6)	16	12-25	526 (17.2)	25	17-39

2+	296 (5.8)	16	11-26.5	270 (8.8)	31	20-45

**Site**^**3**^						

Proximal	1469 (28.6)	13	10-19	1460 (47.4)	21	15-32

Distal	1269 (24.7)	14	10-18	905 (29.6)	22	15-33

Rectum	2236 (43.6)	15	11-21	572 (18.7)	21	14-34

Non specific	159 (3.1)	14	10-22	126 (4.1)	21.5	16-36

**Stage**						

I	575 (11.2)	14	11-20	198 (6.5)	22	14-33

II	1148 (22.4)	14	11-19	703 (23.0)	22	15-34

III	1359 (26.5)	14	11-20	722 (23.6)	21	14-33

IV	639 (12.5)	15	11-21	569 (18.6)	21	15-31

Unknown	1390 (27.1)	14	10-20	856 (28.0)	22	14-33

**Discharge status**						

Public	2703 (59.6)	15	11-21	1835 (69.2)	23	16-34

Private	1836 (40.5)	13	10-18	817 (30.8)	19	13-29

**Discharged to**						

Home	4374 (85.2)	14	10-19	2176 (71.0)	20	14-29

Care	519 (10.1)	18	13-26	472 (14.4)	29	20-42

Death	133 (2.6)	19	11-38	245 (8.0)	26	13-39

Other	107 (2.1)	21	13-32	170 (5.6)	25	11-44

**Same health board**^**4**^						

Yes	4591 (89.4)	14	11-21	2831(92.4)	22	15-33

No	542 (10.6)	14	11-20	232 (7.6)	18.5	13-29.5

**Hospital volume**^**5**^						

< 20	341 (6.6)	14	11-21	242 (7.9)	24	15-34

20-39	904 (17.6)	15	11-20	418 (13.7)	21	15-32

40-59	1592 (31.0)	14	11-20	828 (27.0)	22	16-33

60-79	911 (17.8)	13	10-18	518 (16.9)	21	15-35

80-100	516 (10.1)	14	10-21	344 (11.2)	21	13-34

> 100	869 (16.9)	15	11-21	713 (23.3)	21	14-32

**Surgeon volume**^**6**^						

Low (< 15)	1465 (28.5)	15	11-21	1302 (42.5)	22	15-34

Medium (15-29)	1779 (34.7)	14	11-20	840 (27.4)	22	15-32

High (> 30)	1889 (36.8)	14	10-20	921 (30.1)	21	14-33

^1^SAHRU 2002 index, ^2^Charlson weights for surgery episode excluding cancer diagnosis, ^3^ICD-O 2 topography codes, ^4^patient resides in same health board region as hospital where surgery occurred, ^5^median number of colorectal resections performed at the hospital per year, ^6^median number of colorectal resections performed by the surgeon per year.

Table 2 shows crude and adjusted odds ratios (ORs) for factors predicting prolonged LOS (> 24 days). In the adjusted analysis, for both emergency and elective admissions, likelihood of longer LOS was significantly higher in patients who were older, had co-morbidities and were unmarried; it was reduced for private patients. For emergency admissions only, likelihood of longer LOS was lower for patients admitted to higher-volume hospitals. These factors did not differ notably according to the cut-off used in the sensitivity analyses, (data not shown).

**Table 2:** Factors predicting prolonged LOS in colorectal cancer patients having resection 2002-2008

	Elective Patients LOS > 24 days (n = 5133)					Emergency Patients LOS > 24 days (n = 3063)				
	**Crude**		**Adjusted**			**Crude**		**Adjusted**		

	**OR**^**1**^	**95% CI**	**OR**^**2**^	**95% CI**	**LRT**	**OR**^**1**^	**95% CI**	**OR**^**2**^	**95% CI**^**3**^	**LRT**^**4**^

**Age at diagnosis**										

< 60	1.0		1.0	-		1.0	-	1.0		

60-69	1.60	1.26-2.04	1.55	1.19-2.01	*p = 0.001*	1.89	1.48-2.43	1.56	1.18-2.05	*p < 0.001*

70-79	2.31	1.84-2.91	1.87	1.45-2.43	*p = 0.001*	3.47	2.76-4.37	2.46	1.90-3.18	*p < 0.001*

80+	4.95	3.85-6.36	3.47	2.57-4.66	*p = 0.001*	4.63	3.63-5.90	2.83	2.13-3.76	*p < 0.001*

**Gender**										

Male	1.0	-	-	-	-	1.0	-	-	-	

Female	0.85	0.73-0.99	-	-	-	1.03	0.89-1.19	-	-	

**Marital status**										

Married	1.0	-	1.0			1.0	-	1.0		*p = 0.001*

Other	1.65	1.42-1.92	1.20	1.00-1.43	*p = 0.047*	1.86	1.61-2.15	1.34	1.13-1.59	*p = 0.001*

**Deprivation index**^**5**^										

1 (least deprived)	1.0	-	-	-		1.0	-	-	-	

2	1.06	0.81-1.40	-	-		1.13	0.87-1.47	-	-	

3	0.98	0.74-1.28	-	-		1.25	0.95-1.64	-	-	

4	0.92	0.71-1.19	-	-		1.35	1.06-1.72	-	-	

5 (most deprived)	1.08	0.88-1.34	-	-		1.42	1.16-1.75	-	-	

missing	1.07	0.79-1.45	-	-		-	-			

**Smoking status**										

Ever	1.0	-	1.0			1.0	-	-	-	

Never	0.83	0.70-0.98	0.86	0.71-1.04	*p = 0.005*	0.91	0.73-1.01	-	-	

Unknown	1.32	1.07-1.62	1.28	1.01-1.63	*p = 0.005*	0.94	0.74-1.09	-	-	

**Comorbidity**^**6**^										

0	1.0	-	1.0	-		1.0	-	1.0	-	

1	2.15	1.78-2.60	1.70	1.36-2.12	*p = 0.001*	1.99	1.65-2.41	1.84	1.47-2.31	*p < 0.001*

2+	2.44	1.86-3.19	1.66	1.21-2.27	*p = 0.001*	2.84	2.19-3.68	2.46	1.83-3.31	*p < 0.001*

**Site**^**7**^										

Proximal	1.0	-	1.0			1.0	-	-	-	

Distal	0.98	0.79-1.22	1.06	0.82-1.36	*p = 0.001*	1.01	0.86-1.20	-	-	

Rectum	1.48	1.23-1.77	1.83	1.48-2.25	*p = 0.001*	0.99	0.81-1.20	-	-	

Non specific	1.33	0.86-2.06	1.62	0.98-2.66	*p = 0.001*	1.09	0.76-1.58	-	-	

**Stage**										

I	1.06	0.82-1.38	-	-		1.01	0.73-1.39	-	-	

II	0.87	0.69-1.07	-	-		1.16	0.94-1.43	-	-	

III	1.0	-	-	-		1.0	-	-	-	

IV	1.06	0.83-1.37	-	-		0.87	0.69-1.09	-	-	

Unknown	1.10	0.90-1.35	-	-		1.49	0.95-1.43	-	-	

**Discharge status**										

Public	1.0	-	1.0			1.0	-	1.0		

Private	0.52	0.44-0.62	0.59	0.49-0.71	*p = 0.001*	0.56	0.47-0.67	0.67	0.56-0.81	*p < 0.001*

**Discharged to**										

Home	1.0	-	1.0			1.0	-	1.0	-	

Care	2.60	2.10-3.21	1.76	1.38-2.26	*p = 0.001*	2.93	2.39-3.60	1.96	1.55-2.48	*p < 0.001*

Death	4.89	3.43-6.96	3.00	1.97-4.56	*p = 0.001*	2.01	1.35-2.63	1.08	0.78-1.48	*p < 0.001*

Other	4.43	3.19-7.01	3.40	2.19-5.28	*p = 0.001*	1.93	1.41-2.64	1.73	1.22-2.47	*p < 0.001*

**Same health board**^**8**^										

Yes	1.0		-	-		1.0	-			

No	1.09	0.86-1.39	-	-		0.61	0.46-0.82	0.66	0.48-0.93	*p = 0.016*

**Hospital volume**^**9**^										

< 20	1.12	0.82-1.53	-	-		1.08	0.81-1.43	1.17	0.82-1.67	*p = 0.002*

20-39	0.99	0.79-1.23	-	-		0.88	0.69-1.12	0.95	0.68-1.31	*p = 0.002*

40-59	1.0	-	-	-		1.0	-	1.0	-	

60-79	0.74	0.59-0.94	-	-		0.93	0.74-1.16	0.63	0.46-0.85	*p = 0.002*

80-100	1.25	0.96-1.61	-	-		0.77	0.60-1.00	0.56	0.39-0.80	*p = 0.002*

> 100	1.20	0.97-1.49	-	-		0.80	0.65-0.98	0.74	0.53-1.02	*p = 0.002*

**Surgeon volume**^**10**^										

Low (< 15)	1.0	-	-	-		1.0	-	-	-	

Medium (15-29)	0.76	0.63-0.92	-	-		0.91	0.76-1.09	-	-	

High (> 30)	0.83	0.69-0.99	-	-		0.89	0.75-1.05	-	-	

^1^unadjusted odds ratio, ^2^adjusted for variables shown and hospital health board, ^3^95% confidence intervals, ^4^p-values from likelihood ratio tests, ^5^SAHRU 2002 index, ^6^Charlson weights for surgery episode excluding cancer diagnosis, ^7^ICD-O 2 topography codes, ^8^patient resides in same health board region as hospital where surgery occurred, ^9^median number of colorectal resections performed at the hospital per year, ^10^median number of colorectal resections performed by the surgeon per year

**Figure 3:**
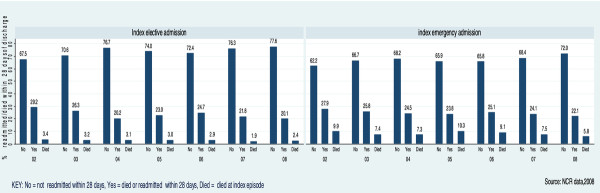
**28 day emergency readmission rates by index episode admission type and year**.

Of 8197 patients who had a resection, 394 (4.8%) died in hospital before discharge. Of the remainder (n = 7803), 111 (0.9%) died within the following 28 days. 25.1% (n = 1959) were readmitted within 28 days, 16.7% (n = 1302) electively and 8.4% (n = 657) as emergencies. The most common principal diagnoses amongst emergency readmissions were: cancer 20.9% (n = 120); surgical complications including post-operative wound infections 14.2% (n = 82); obstruction 7.6% (n = 44); and urinary tract infection 4.8% (n = 28). Readmission rates did not change over the period of the study (see Figure 3).

In the adjusted analysis, the factors significantly associated with increased risk of emergency readmission were increased deprivation and comorbidity, later stage disease, being a public patient and having had an index emergency admission; older patients were less likely to be readmitted (Table 3). After adjusting for these factors, LOS was significantly associated with readmission risk, and individuals in the upper quartile of LOS were 67% more likely to be readmitted.

**Table 3:** Factors predicting emergency readmission/death at < 29 days of discharge from index surgery episode

	Emergency readmissions		Crude		Adjusted		
	**No** **n = 7146(%)**	**Yes** **n = 657(%)**	**OR**^**1**^	**95% CI**^**3**^	**OR**^**2**^	**95% CI**^**3**^	**LRT**^**4**^

**Age at diagnosis**							

< 60 years	1732 (91.3)	164 (8.6)	1.0	-	1.0		

60-69 years	2031 (93.2)	149 (6.8)	0.77	0.61-0.98	0.70	0.54-0.90	*p = 0.038*

70-79 years	2267 (91.1)	222 (8.9)	1.03	0.84-1.28	0.76	0.60-0.97	*p = 0.038*

80+	1116 (90.1)	122 (9.9)	1.15	0.90-1.48	0.78	0.58-1.05	*p = 0.038*

**Gender**							

Male	4093 (91.4)	385 (8.6)	1.0	-	-	-	

Female	3053 (91.82)	272 (8.18)	0.95	0.80-1.11	-	-	

**Marital status**							

Married	4235 (91.81)	378 (8.19)	1.00	-	-	-	

Other	2888 (91.25)	277 (8.75)	1.07	0.91-1.26	-	-	

**Deprivation index**^**5**^							

1 (least deprived)	1355 (93.5)	94 (6.5)	1.00	-	1.0	-	

2	923 (91.7)	83 (8.3)	1.30	0.95-1.76	1.26	0.90-1.78	*p = 0.060*

3	895 (92.1)	77 (7.9)	1.24	0.91-1.69	1.25	0.88-1.77	*p = 0.060*

4	1213 (91.6)	111 (8.4)	1.32	0.99-1.75	1.20	0.87-1.66	*p = 0.060*

5 (most deprived)	2193 (90.7)	225 (9.3)	1.48	1.15-1.90	1.42	1.07-1.90	*p = 0.060*

missing	567 (89.4)	67 (10.6)	1.70	1.22-2.36	1.68	1.16-2.42	*p = 0.060*

**Smoking status**							

Ever	2552 (91.4)	239 (8.6)	1.0		-	-	

Never	3170 (91.8)	283 (8.2)	0.95	0.80-1.14	-	-	

Unknown	1318 (91.1)	129 (8.9)	1.04	0.83-1.31	-	-	

**Comorbidity**^**6**^							

0	5726 (92.2)	482 (7.8)	1.0	-	1.0		*p < 0.001*

1	1013 (90.6)	105 (9.4)	1.23	0.99-1.54	1.10	0.86-1.44	*p < 0.001*

2+	407 (85.3)	70 (14.7)	2.04	1.56-2.68	1.83	1.36-2.46	*p < 0.001*

**Site**^**7**^							

Proximal	2516 (91.6)	231 (8.4)	1.00	-	-	-	

Distal	1910 (91.9)	168 (8.1)	0.96	0.78-1.18	-	-	

Rectum	2480 (91.4)	233 (8.6)	1.02	0.85-1.24	-	-	

Non specific	240 (90.6)	25 (9.4)	1.13	0.73-1.75	-	-	

**Stage**							

I & II	2338 (92.5)	190 (7.5)	1.0	-	1.0	-	

III & IV	2856 (90.6)	295 (9.4)	1.27	1.05-1.54	1.22	1.00-1.51	*p = 0.086*

Unknown	1951 (91.9)	172 (8.1)	1.08	0.87-1.34	1.00	0.78-1.27	*p = 0.086*

**Index admission**							

Elective	4623 (92.7)	367 (7.4)	1.0		1.0	-	

Emergency	2522 (89.7)	290 (10.3)	1.45	1.23-1.70	1.21	1.00-1.46	*p = 0.052*

**Discharge status**							

Public	3888 (90.6)	404 (9.4)	1.0		1.0	-	

Private	2406 (93.8)	160 (6.2)	0.64	0.53-0.77	0.75	0.61-0.92	*p = 0.008*

**Discharged to**							

Home	6032 (92.2)	508 (7.8)	1.0	-	1.0		

Care	888 (89.7)	102 (10.3)	1.36	1.09-1.71	1.16	0.90-1.51	*p < 0.001*

Other	224 (84.2)	42 (15.8)	2.23	1.58-3.13	2.13	1.48-3.08	*p < 0.001*

**Same health board**^**8**^							

Yes	6451 (91.5)	603 (8.6)	1.0	-	-	-	

No	695 (92.8)	54 (7.2)	0.83	0.62-1.11	-	-	

**Hospital volume**^**9**^							

< 20	482 (88.1)	65 (11.9)	1.59	1.18-2.14	-	-	

20-39	1134 (90.0)	126 (10.0)	1.31	1.03-1.66	-	-	

40-59	2120 (92.2)	180 (7.8)	1.0	-	-	-	

60-79	1232 (91.8)	110 (8.2)	1.05	0.82-1.35	-	-	

80-100	753 (91.4)	71 (8.6)	1.11	0.83-1.48	-	-	

> 100	1425 (93.1)	105 (6.9)	0.86	0.67-1.22		-	

**Surgeon volume**^**10**^							

Low (< 10)	2360 (90.8)	238 (9.2)	1.0	-	-	-	

Medium (10-29)	2292 (91.9)	201 (8.1)	0.87	0.71-1.06	-	-	

High (> 30)	2494 (92.0)	218 (8.0)	0.87	0.71-1.05	-	-	

**Surgery LOS quartile**							

Q1	2269 (93.5)	157 (6.5)	1.0	-	1.0	-	

Q2	1553 (92.8)	121 (7.2)	1.12	0.88-1.44	1.16	0.89-1.51	*p < 0.001*

Q3	1651 (92.0)	143 (8.0)	1.25	0.99-1.58	1.17	0.90-1.53	*p < 0.001*

Q4	1673 (87.6)	236 (12.4)	2.04	1.65-2.52	1.67	1.29-2.16	*p < 0.001*

^1^unadjusted odds ratio, ^2^adjusted for variables shown and hospital health board, ^3^95% confidence intervals, ^4^p-values from likelihood ratio tests, ^5^SAHRU 2002 index, ^6^Charlson weights for surgery episode excluding cancer diagnosis, ^7^ICD-O 2 topography codes, ^8^patient resides in same health board region as hospital where surgery occurred, ^9^median number of colorectal resections performed at the hospital per year, ^10^median number of colorectal resections performed by the surgeon per year

## Discussion

### Strengths and limitations

This study is based on high-quality cancer registration data, providing confidence that the patients included had colorectal cancer. The study is population-based and provides - for the first time - detailed information on factors predicting LOS for patients admitted both as elective and emergencies; the latter group account for over one-third of colorectal cancers undergoing resection. Their longer average LOS demonstrates the major impact that they are likely to have on healthcare costs in Ireland and elsewhere.

Just over 6% of cases recorded by NCR as having a resection in a public hospital had no corresponding HIPE record. Failure to find a match can occur for several reasons including: typographical errors in fields used for matching, missing data on either system, or no mention of cancer on the HIPE record, in which case the record would not be made available to NCR. The missing episodes were distributed across hospitals and years and are unlikely to be a cause of bias.

### Factors associated with LOS

Patient-related factors, including age, higher levels of co-morbidities and marital status, were associated with increased risk of lengthy LOS for both emergency and elective admissions. It is expected that older and sicker patients would have a longer LOS. The observation that married patients have shorter LOS may reflect a lack of social support among unmarried patients [27,28]. Grocott et al. [29] observed that a shortage of step-down beds can lead to patients occupying hospital beds for longer than required. Our finding of prolonged LOS among patients who were not married or were discharged to care supports this.

While total pre- and post-operative LOS is the most relevant outcome as regards costs to the healthcare system, investigation of pre- and post-operative LOS may also be informative. We found emergency patients waited longer for surgery than elective patients and stayed longer post surgery. Emergency patients are likely to require more care and investigation to establish a definitive diagnosis before treatment which probably explains their higher pre-surgery LOS and overall median LOS. Based on 2008 figures, an approximate average inpatient cost per night for patients having bowel surgery in Ireland is €930 (HSE Casemix/HIPE Unit Ready Reckoner 2010 - Diagnostic Related Group- G02B), and while this figure may reduce as patients recover post-operatively, any reduction in LOS could generate significant cost savings.

### Emergency admissions

The proportion of colorectal resections conducted as an emergency procedure in this study (37%) was higher than rates reported in England (32.5%) [30]. The difference between Ireland and England may be explained by the mixed public-private healthcare system in Ireland, compared to the almost entirely public system in England; emergency patients are unlikely to be treated in private hospitals and will thus be over-represented among the public hospitals covered by HIPE. The high proportion of emergency admissions may be partly due to a lack of knowledge of colorectal symptoms among the public [31,32]. Alternatively, those who present as emergencies may have had symptoms but delayed seeing a primary care doctor because of denial or fear, or may have been misdiagnosed or inadequately investigated [33]. A further possible explanation is system delays, for example, long waiting lists for colonoscopies. This is a recognised issue in the public health system in Ireland: in April 2009 more than 2,300 individuals had been waiting for a colonoscopy for more than three months [34]. In this context, it is possible that clinicians may admit patients as emergencies to overcome shortages in access to investigations as outpatients or elective surgery time; we do not have any information on the likely extent of this practice.

Patients admitted as emergencies were older, sicker and presented with later stage cancer suggesting this group may have poorer access to appropriate care for the reasons outlined above. Our results also show emergency admissions have poorer outcomes, in that they are more likely to die at index and in the 28 days after hospital discharge. Similar reports in the UK resulted in a call for population-based screening to increase awareness and reduce the number of emergency admissions [35]. A population-based colorectal cancer screening programme will be introduced in Ireland in 2012 [36]. An important component of its evaluation should be to examine trends in emergency admissions.

The high proportion of emergency admissions, coupled with evidence of increased length of stay and poorer outcomes, has significant cost implications for the Irish health service. This is likely to be true internationally and has already been documented in Australia [37].

### Readmissions

Unplanned readmission rates after colorectal surgery are considered a marker of quality of surgical care and numerous studies have attempted to identify predictors of early readmission with mixed results [38]. In England, Faiz et al. [12] reported 28 day readmission rates ranging from 7.7% to 8.6% for colon surgery and 8.8% to 11.9% for rectal surgery during 2001-2006, figures comparable with our rate of 7.3%. Comparisons between studies are difficult however because of differences in time frames for readmission, patient groups, and in whether planned readmissions were included.

One of the concerns about initiatives to reduce LOS is that they might lead to increased readmission [11,12]. Some studies have found no association between LOS and risk of readmission [39,40]. We, by contrast, found a positive relationship: longer initial LOS increased risk of emergency readmission, which is plausible since these were older, sicker patients initially.

### Comparisons of LOS between countries

Differences in median LOS between studies must be considered within the context of health care organization and initiatives to reduce LOS. In this study, undertaken within a mixed public-private healthcare system with no national initiatives in place, the median LOS was higher than figures from similar studies in the US and UK. Faiz et al. reported a median LOS of 11 and 13 for colon and rectal resection respectively in English NHS trusts [12] based on elective admissions and including non-cancer resections. When limited to patients with colorectal malignancies, the range 11-14 days for colectomy procedures and 13-15 for rectal procedures are comparable with our figures for elective admissions. In the US, Leung et al. reported a median post-operative LOS of 8 days in 183 veteran patients, 118 of whom had cancer [25]. There was no breakdown by cancer site or stage so comparison with our results is difficult. Although Leung et al. noted that the patients were not subject to pressure from insurers to decrease LOS, since they were recruited from a veterans' hospital, the comparison with our results (with regard to shorter LOS in private patients) and those with Faiz et al. (which relate to an entirely public system), suggests some aspects of private healthcare provision result in shorter LOS. Whether these influences are due to differences in case-mix and complexity or health system a factor are unclear, and warrants further investigation.

### Time trends in LOS

In common with patterns in England and the USA [12,41] we observed a modest decrease in median LOS over time, and since 2006 in particular. This may be due to the recent trend towards greater specialisation of cancer care in Ireland [42]. There has been an emphasis on re-organisation of rectal cancer surgery and increased use of laparoscopic rectal procedures. We were unable to distinguish between open and laparoscopic procedures in our data, so were unable to formally investigate the extent to which increased use might account for the observed trend. Nor did we have any information on whether individual units were running enhanced recovery programmes or using specialised laparoscopic colonic surgery both of which might tend to shorten LOS.

As part of a national clinical strategy in Ireland the Elective Surgery Programme was launched in 2010 with the aim of improving the patient's elective surgical journey. It includes the development of integrated care pathways http://www.hse.ie/eng/about/Who/clinical/. Our study provides a baseline against which the impact of the programme on LOS could be evaluated.

## Conclusion

One quarter of patients stay in hospital at least 25 days following colorectal resection. More than one-third of resected patients are admitted as emergencies and this group has a significantly longer median LOS. After adjusting for clinical factors, several patient- and health-service related factors influenced the likelihood of longer LOS. Longer LOS was associated with increased risk of emergency readmission within 28 days. The cost implications of these findings for the health services in Ireland and elsewhere are significant and further development of strategies to reduce emergency admissions or LOS would be valuable.

## Competing interests

The authors declare that they have no competing interests.

## Authors' contributions

MK carried out the analysis and wrote the initial drafts of the manuscript. LS conceived the study, provided statistical support and helped draft the manuscript. FD and TK linked the data. LS and HC helped with interpretation of the data and results. All authors contributed to the final draft of the manuscript. All authors read and approved the final manuscript.

## Pre-publication history

The pre-publication history for this paper can be accessed here:

http://www.biomedcentral.com/1472-6963/12/77/prepub
